# Hemopexin induces neuroprotection in the rat subjected to focal cerebral ischemia

**DOI:** 10.1186/1471-2202-14-58

**Published:** 2013-06-10

**Authors:** Beibei Dong, Min Cai, Zongping Fang, Haidong Wei, Fangyun Zhu, Guochao Li, Hailong Dong, Lize Xiong

**Affiliations:** 1 Department of Anesthesiology, Xijing Hospital, Fourth Military Medical University, Xi’an, Shanxi 710032, China; 2 Key Laboratory of the Critical Care Medicine of PLA, Xijing Hospital, the Fourth Military Medical University, Xi’an, Shanxi 710032, China

**Keywords:** Hemopexin, Cerebral ischemia, Neuroprotection, Intracerebroventricular injection, Endogenous protection

## Abstract

**Background:**

The plasma protein hemopexin (HPX) exhibits the highest binding affinity to free heme. In vitro experiments and gene-knock out technique have suggested that HPX may have a neuroprotective effect. However, the expression of HPX in the brain was not well elucidated and its expression after cerebral ischemia-reperfusion injury was also poorly studied. Furthermore, no in vivo data were available on the effect of HPX given centrally on the prognosis of focal cerebral ischemia.

**Results:**

In the present study, we systematically investigated expression of HPX in normal rat brain by immunofluorescent staining. The results showed that HPX was mainly expressed in vascular system and neurons, as well as in a small portion of astrocytes adjacent to the vessels in normal rat brain. Further, we determined the role of HPX in the process of focal cerebral ischemic injury and explored the effects of HPX treatment in a rat model of transient focal cerebral ischemia. After 2 h’ middle cerebral artery occlusion (MCAO) followed by 24 h’ reperfusion, the expression of HPX was increased in the neurons and astrocytes in the penumbra area, as demonstrated by immunohistochemistry and Western blot techniques. Intracerebroventricular injection of HPX at the onset of reperfusion dose-dependently reduced the infarct volumes and improved measurements of neurological function of the rat subjected to transient focal cerebral ischemia. The neuroprotective effects of HPX sustained for up to 7 days after experiments.

**Conclusions:**

Our study provides a new insight into the potential neuroprotective role of HPX as a contributing factor of endogenous protective mechanisms against focal cerebral ischemia injury, and HPX might be developed as a potential agent for treatment of ischemic stroke.

## Background

Stroke is the second leading cause of death worldwide and the second leading cause of long-term disability in developed countries. Demographic data has shown that 800,000 individuals suffered from stroke each year in the United States, among which, approximately 150,000 died [1]. In China, the figures were even more striking. About 1.5 to 2 million new strokes occurred each year in China, among which more than 1 million died [2]. A number of therapeutic strategies have been developed for treating stroke over the past several decades, such as hypothermia, tissue plasminogen activator (tPA) and ischemic preconditioning. However, when one considers the narrow therapeutic window, the limited clinical efficacy, and the inevitable side effects associated with such therapeutic tools, the outcomes are far from satisfactory. For this reason, it is necessary to explore new strategies for the clinical management of this potentially fatal disease.

Studies have shown that many mechanisms are associated with cerebral ischemia-reperfusion injury, including excitotoxicity, brain edema, and neuronal apoptosis [3,4]. Additionally, free heme is released from methemoglobin after an occurrence of cerebral ischemia-reperfusion, and as such, represents a toxic component in the peripheral blood [5].

Hemopexin (HPX) is a 60 kDa serum glycoprotein with the greatest binding affinity (*K*_*d*_< 1 pM*)* to heme [6]. Many types of cells synthesize HPX including hepatic parenchymal cells, ganglionic and photoreceptor cells of the retina, kidney mesangial cells, and cells in the peripheral nervous system [7,8]. HPX behaves as an efficient scavenger of heme in overloaded peripheral blood [5,9]. Surprisingly, HPX was reported to be locally expressed in the central nervous system (CNS) [7,10], but reports on its cellular localization in CNS are contradictory and the temporal changes of its expression after cerebral ischemia has not be observed. In addition, prior work found that HPX-null mice sustained greater infarct volumes and behavioral deficits than their wild-type counterparts in a model of transient middle cerebral artery occlusion, indicating its neuroprotective role against stroke-related damage [10]. However, it remained unclear whether administration of exogenous HPX can improve the outcome of cerebral ischemia-reperfusion injury.

In the present study, we designed experiments to determine the cellular location and expression level changes of HPX after focal ischemia and reperfusion injury, and subsequently explore the therapeutic effect of HPX using a rat model of middle cerebral artery occlusion (MCAO).

## Methods

### Animals

The experimental procedures in this study were approved by the Ethics Committee for Animal Experimentation of the Fourth Military Medical University, China. Male Sprague–Dawley (SD) rats, with a weight of approximately 280–320 g, were provided by the Experimental Animal Center of the Fourth Military Medical University. Animals were housed under environmental controlled conditions, with a 12-hour light/dark cycle, an ambient temperature of 24 ± 2°C and free access to food and water. We made efforts to minimize the number of animals used and their suffering.

### Experiment design

#### Experiment I

The objective of this experiment was to examine localization of hemopexin in normal rat brain and to explore its protein level changes in the ischemic penumbra following ischemia-reperfusion. The rats were randomly assigned to the group subjected to middle cerebral artery occlusion (MCAO) or a sham group. Rats in the MCAO group experienced ischemia for 2 h. Next, at 2 h, 6 h, 12 h and 24 h after reperfusion (n=4 rats per group at each time point), the frequency of HPX positive cells in brain sections and the levels of HPX protein in brain homogenates were assessed by immunohistochemistry and Western immunoblotting, respectively.

#### Experiment II

The objective of this experiment was to evaluate the neuroprotective effects of varying doses of HPX on transient focal cerebral ischemia following intracerebroventricular injection. In this experiment, 50 rats were randomly assigned to 5 groups: control group; vehicle group (intracerebroventricularly injected with 0.1% sodium azide); and dose-dependently delivered HPX injection groups, in which rats received an intracerebroventricular injection of HPX at the following doses: 0.46 mg/ml (low dose), 0.93 mg/ml (middle dose) and 1.86 mg/ml (high dose). Animals were dose-dependently exposed to 5 μl of rat hemopexin reference serum (ICL, Portland, OR, USA) or the carrier vehicle (5 μl, 0.1% sodium azide) at the beginning of reperfusion using a single intracerebroventricular injection following a previously published protocol [11]. Immediately prior to injection, HPX was dissolved in 0.1% sodium azide and diluted to a final concentration in normal saline.

#### Experiment III

The objective of this experiment was to assess the long-term therapeutic effects of HPX in the setting of transient focal cerebral ischemia. In this experiment, 16 rats were randomly selected into a group receiving the carrier vehicle alone, or a group receiving the test agent HPX (n=8 rats per group). All animals were subjected to MCAO. Both HPX (1.86 mg/ml, 5 μl) and the carrier vehicle (0.1% sodium azide, 5 μl) were administered to the animals by the intracerebroventricular route as soon as the reperfusion procedure had been initiated. A qualified observer, who was blind to the grouping information, evaluated the neurological behavior of the rats daily. These evaluations were measured after MCAO until day seven after administration of HPX, and done according to an 18-point scoring system [12]. Following decapitation of the rats, the brain infarct volumes were assessed.

### Western immunoblot analysis

Rats (n = 4 per group, per time-point) were deeply anesthetized before decapitated, and brain tissues quickly removed from the ipsilateral peri-ischemic cortex (ischemic penumbra) in alignment with the corresponding time-points after ischemia-reperfusion and frozen for subsequent tissue homogenization. Protein extracts were obtained by grinding tissue in RIPA buffer containing protease inhibitors. An equal amount of protein (40 μg) was loaded into each lane of a polyacrylamide-SDS gel, subjected to electrophoresis and resolved proteins transferred to a PVDF membrane. Membranes were blocked in 5% BSA and incubated with the appropriate primary antibodies overnight at 4°C. The primary antibodies used in this procedure were; anti-HPX (1:500 dilution, Santa Cruz Biotechnology Inc., Santa Cruz, CA, USA) and an anti-rabbit β-actin antibody (1:1000 dilution, CWBIO, Peking, China), which was used as an internal protein loading control. The membranes were washed three times in TBST (Tris-Buffered Saline Tween-20) for 5 min per each, then incubated for 1 h at room temperature with the appropriate goat anti-rabbit secondary antibodies (1:1000 dilution, CWBIO, Peking, China). To visualize specific protein bands, the immunoblots were immersed in enhanced chemiluminescent (ECL) reagent, followed by exposure to ECL- Hyperfilm (Amersham Biosciences, Piscataway, NJ, USA).

### Immunofluorescence staining

Animals were first anesthetized with an intraperitoneal administration of 40 mg kg^− 1^ pentobarbital sodium, then transcardially perfused with saline followed immediately by 4% paraformaldehyde in 100 mM phosphate buffer (pH 7.4). Subsequently the brains were removed and immersed in 20% sucrose in phosphate-buffered saline (PBS) overnight at 4°C. Brains were then transferred to a 30% sucrose solution for 24 h. Corneal slices were prepared as 12 um sections using a standard cryostat, and stored at −20°C. Sections were blocked in 10% normal goat serum supplemented with 0.3% Triton X-100 for 1 h at room temperature. Sections were next incubated with a combination of primary anti-mouse glial fibrillary acidic protein (GFAP, at a dilution of 1:500; Sigma-Aldrich, Madrid, Spain) and a primary rabbit polyclonal antibody directed against HPX (at a dilution of 1:100; Santa Cruz Biotechnology Inc., Santa Cruz, CA, USA) at 4°C overnight. Sections were next washed three times in PBS, then incubated in NeuroTrace (at a dilution of 1:1000; Molecular Probes, Eugene, OR, USA) or with two secondary antibodies for 2 h at room temperature. Secondary antibodies were: donkey anti-mouse conjugated to green-fluorescent Alexa Fluor 488 (at a concentration of 1:500; Vector Laboratories, Inc., Burlingame, CA) and donkey anti-rabbit conjugated to red-fluorescent Alexa Fluor 594 (at a dilution of 1:500; Vector Laboratories). Sections were incubated with DAPI (1 ng/μl; Sigma-Aldrich, Poole, UK) to stain the nuclei for 5 min at room temperature. Fluorescent signals were detected by confocal laser scanning microscopy (Olympus, FV1000).

### Focal cerebral ischemia

**Figure 1:**
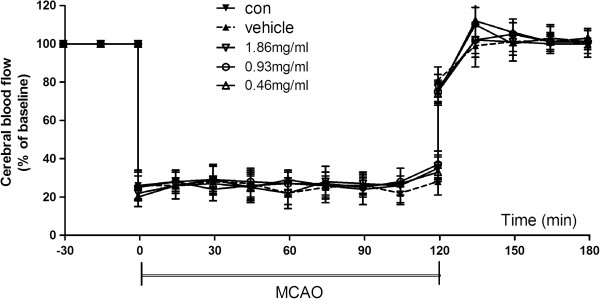
**Regional cerebral blood flow of ischemic hemisphere during MCAO surgery.** Control animals suffered from middle cerebral artery occlusion (MCAO) without any treatment; others received an intracerebroventricular injection with vehicle (0.1% sodium azide) or HPX at the following doses: 0.46 mg/ml (low dose), 0.93 mg/ml (middle dose) and 1.86 mg/ml (high dose). Data are expressed as mean±SEM.

Focal cerebral ischemia was induced in rats by MCAO using an intraluminal filament technique, as previously described [13,14]. Briefly, rats were fasted for 12 h prior to surgery but were allowed free access to water. Anesthesia was induced by intraperitoneal injection of pentobarbital sodium (40 mg/kg in normal saline). Next, the right common carotid artery (CCA) and the right external carotid artery (ECA) were exposed through a ventral midline neck incision, and were ligated proximally. A 4/0 monofilament nylon suture (Beijing Sunbio Biotech Co., Ltd.) was inserted through an arteriectomy in the common carotid artery just below the carotid bifurcation and into the internal carotid artery approximately 18–20 mm distal to the carotid bifurcation until mild resistance was felt [15]. Next, the middle cerebral artery was occluded. After 2 h of ischemia, the reperfusion process was accomplished by withdrawing the suture then reapplying the suture to the wound. During this process cerebral blood flow was monitored through a disposable fiber optic probe (with a diameter of 0.5 mm) connected to a laser Doppler unit (PeriFlux 5000, Perimed AB, Stockholm, Sweden). Rats that showed more than a 70% reduction in cerebral blood flow were retained in their corresponding groups for data recording (Figure 1). Body temperature of the rats was maintained at 37 ± 0.5°C during the procedure.

### Intracerebroventricular injection

Anesthetized rats were placed on a stereotaxic apparatus and four stainless-steel screws were secured to the skull and occluded. An incision to the scalp exposed the surface of the skull and bregma. A burr hole was drilled into the bone of the right hemisphere with a stainless steel 26-gauge cannula, located 1.5 mm lateral to, and 0.8 mm posterior to the bregma. A 5 μl Hamilton syringe was introduced slowly to 3.5 mm beneath the dural surface to allow dose dependent exposure of rats to 5 μl of rat hemopexin reference serum HPX (ICL, Portland, OR) or vehicle (0.1% sodium azide) by injection. The injection needle was maintained in situ for 5 min before withdrawal.

### Neurobehavioral evaluation and assessment of infarct

At 24 h (or 2, 3, 4, 5, 6 and 7d) after reperfusion, the neurologic behavior of the rats was determined by a qualified observer blinded to the rat grouping strategy, and according to a previously described 18-point scoring system [12]. Rats were anesthetized, decapitated, and the brains were removed, sliced into six separate 2 mm thick coronal sections and stained with a 2% solution of 2,3,5-triphenyltetrazolium chloride (TTC, Sigma, St. Louis, MO) at 37°C for 10 min [13,14]. The slices were then fixed in 4% formalin prior to digital photographic images being recorded (Canon, Tokyo, Japan). Unstained areas were defined as infarcts and were measured by image analysis software (Adobe Photoshop, Version CS3). To exclude the effect of ischemia-induced cerebral edema on the infarct size, the percent of the infarct volume was calculated indirectly according to the formula: ([total contralateral hemispheric volume] - [total ipsilateral hemispheric stained volume]) / (total contralateral hemispheric volume) × 100.

### Statistical analyses

Statistical analyses were made using the SPSS 14.0 data analysis software program. All values, with the exception of the neurologic scores, were described as mean ± SEM, and were analyzed by one-way analysis of variance (ANOVA). Differences between two groups were detected by applying Tukey’s post-hoc test. The neurological scores were described as the median (interquartile range), and these data sets were compared by applying the Kruskal-Wallis test followed by the Mann–Whitney U-test and Bonferroni’s post-hoc correction. Statistical significance was defined as attaining at least a value of P< 0.05.

## Results

### Cellular location of HPX in the normal rat brain

**Figure 2:**
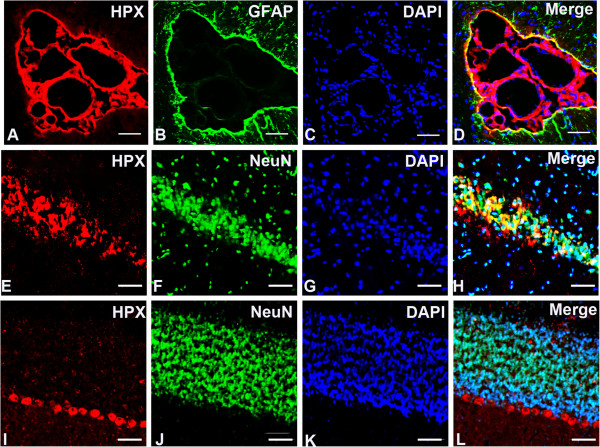
**Immunofluorescence double labeling of HPX (red) and NeuN/GFAP (green) in normal rat brain.** Microphotographs showing the expressions of HPX in the ependymal epithelial cells and choroid plexus cells **(A-D)**, Purkinje cells in the hippocampal neurons **(E-H)**, and cerebellar cortical **(I-L)**. Scale bars = 20 μm.

**Figure 3:**
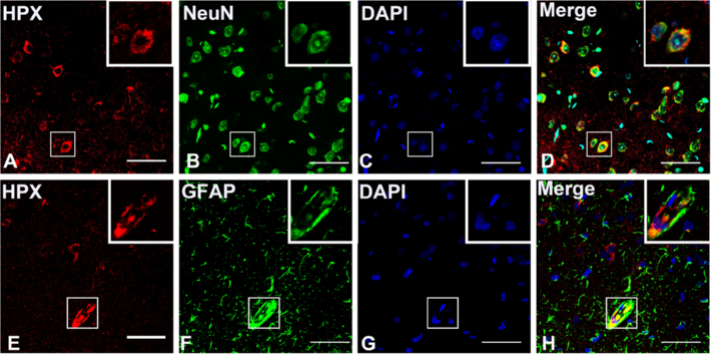
**Localication of HPX in neurons and astrocytes in the cerebral cortex.** HPX was stained in red and NeuN/GFAP was stained in green. Note that HPX staining was strong in the cytoplasm of neurons but was weak in the nucleus **(A-D,E-H)**, and the immunoreactive astrocytes were mainly located near the blood vessels (**E-H**). Scale bars = 40 μm.

Our immunofluorescence staining results demonstrated that the HPX protein was strongly expressed in the cerebral vascular system, especially in the ependymal epithelial cells and choroid plexus cells (Figure 2A-D). In brain parenchyma, HPX immunoreactivities were mainly localized in neurons, predominantly in the hippocampal pyramidal cells, and cerebellar Purkinje cells, as demonstrated by doubling staining of HPX and NeuN, a neuronal marker (Figure 2E-L). The majorities of astrocytes were not stained by HPX except those in the periphery of ventricular system and blood vessels, as shown by doubling staining with glial fibrillary acidic protein (GFAP), a marker for astrocytes (Figure 2A-D; Figure 3E-H).

### Alteration of HPX expression following cerebral ischemia and reperfusion

**Figure 4:**
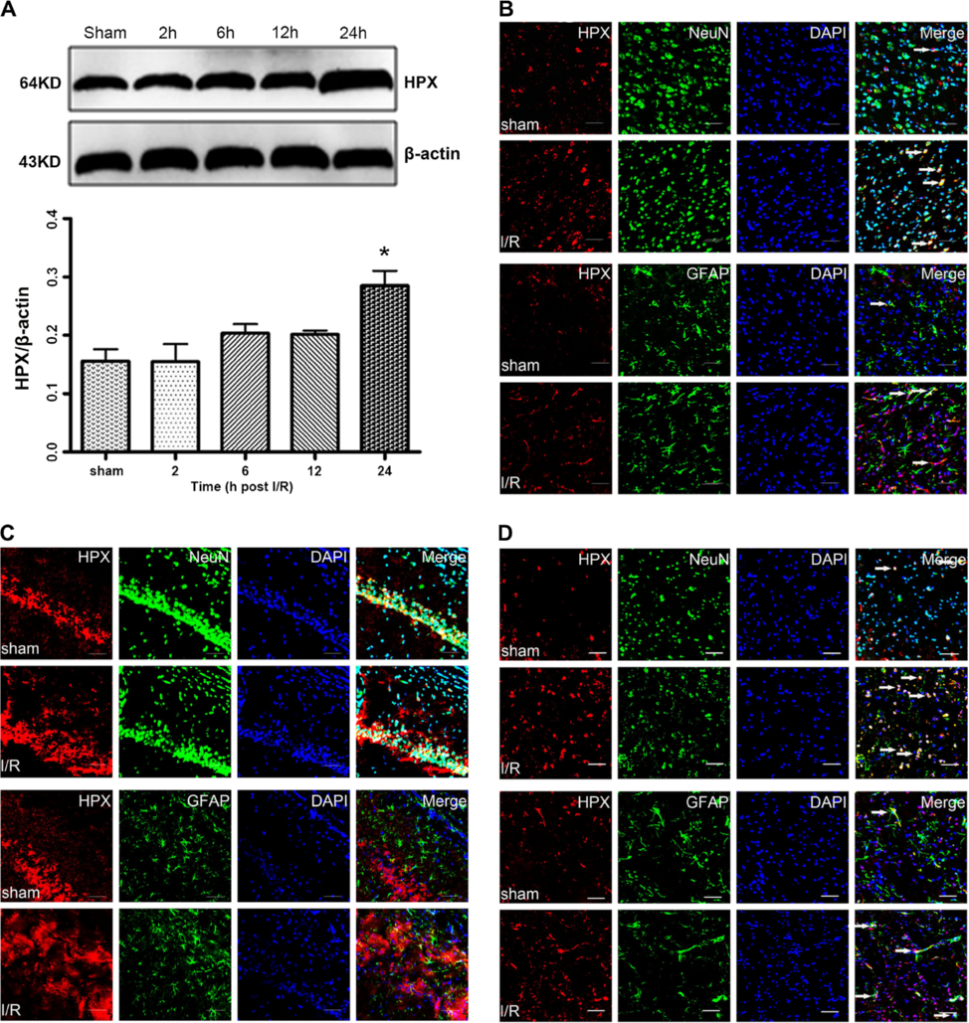
**HPX expression was evaluated in the ischemic penumbra at different time points following reperfusion. A**, Western blotting of the HPX expression in sham and MCAO rats at 2 h, 12 h and 24 h after reperfusion. The expression of β-actin served as a loading control of the protein samples. The relative density of HPX protein to β-actin protein expression among groups was plotted. Data represent mean ± SEM. *P*<* 0.05 vs. Sham group. (n =4 for each group) Double immunofluorescence staining of HPX and NeuN/GFAP indicated that HPX expression in the cortex **B**, hippocampus **C**, and striatum **D**, were notably increased in the I/R group. Scale bars = 20 μm.

Following transient MCAO, the protein levels of HPX were gradually increased in ipsilateral ischemic penumbra, as detected by Western immunoblotting. By 24 h after reperfusion, HPX protein levels in the ischemia-reperfusion (I/R) group increased markedly in comparison to the sham group (P<0.05, Figure 4A). Consistent with the Western immunoblotting findings, the number of HPX positive cells visualized by immunohistochemical staining in the vicinity of ischemic penumbra of the I/R group were notably increased in the cerebral cortex, hippocampus, and striatum (Figure 4B, 4C and 4D).

### Effects of intracerebroventricular injection of HPX on MCAO injuries

**Figure 5:**
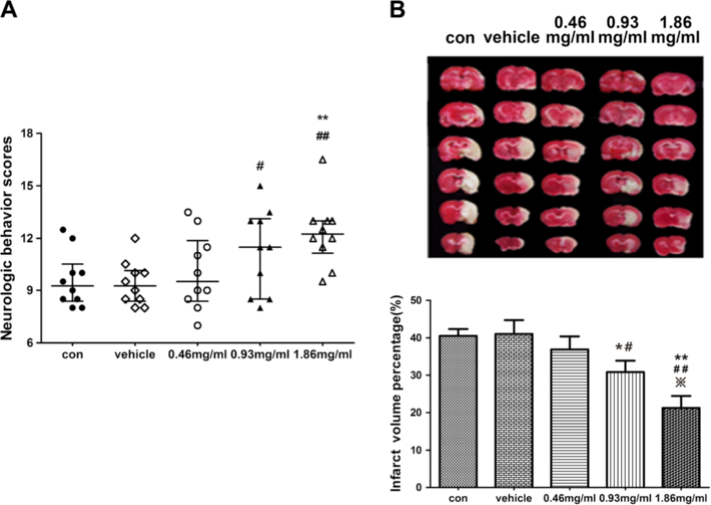
**Neurological deficit (A) and infarct volume percentages (B) of the rats at 24h after ischemia-reperfusion.** Neurological deficit scores were shown as the median (inter-quartile range). Infarct volume percentages were expressed as mean ± SEM. Treatment with HPX improved neurologic scores and reduced brain infarct volumes in a dose-dependent manner. ** P<0.01 in comparison with the control group; ## P*<*0.01 in comparison with the vehicle group; * P<0.05 in comparison with the control group; #P*<*0.05 in comparison with the carrier vehicle group; **※**P*<*0.05 in comparison with the medium-dose group. (n=10 for each group).

Intracerebroventricular administration of different doses of HPX (high dose: 1.86 mg/ml; medium dose: 0.93 mg/ml; low dose: 0.46 mg/ml) improved neurological deficit (A) and infarct volume percentages (B) in a dose-dependent manner by 24 h after ischemia-reperfusion. The neurobehavioral outcome of the high-dose HPX (1.86 mg/ml) administered group was markedly improved as compared with the control and vehicle-given groups (P<0.01 for both comparisons, Figure 5A) at 24 h post-reperfusion. Medium-dose HPX (0.93 mg/ml) also induced an improved neurobehavioral outcome as compared with the vehicle group (P<0.05, Figure 5A). Intracerebroventricular injection of high-dose (1.86 mg/ml) and medium-dose (0.93 mg/ml) HPX also reduced the infarct size in the brain at 24 h post-reperfusion (Figure 5B). Further, the brain infarct volume percent in the high-dose HPX group was also lower than that seen in the medium-dose group (P<0.05, Figure 5B). In view of the best neuroprotective effect, we selected the high-dose concentration of HPX (1.86 mg/ml) as the dose used in the following studies.

### Long-term therapeutic effects of HPX on transient focal cerebral ischemia

**Figure 6:**
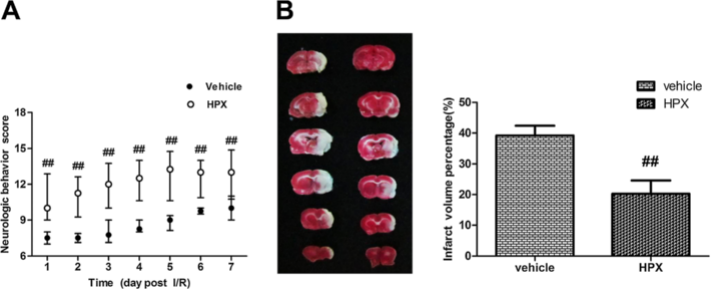
**Neurological deficit scores (A) and infarct volume percentages (B) following transient MCAO over seven days.** The neurobehavioral outcome in the HPX-administration (1.86 mg/ml) group was far superior as compared with the vehicle group. In addition, the brain infarct volumes were significantly reduced. ## *P<*0.01 in comparison with the vehicle group. (n=8 for each group).

The long-term beneficial effects of treatment with high-dose HPX were verified according to the neurobehavioral and infarct volume data within 7d following MCAO (Figure 6). Neurologic behavioral scores were recorded daily after MCAO until day 7 when animals were decapitated, and the percent of brain volumes were determined. Neurological behavior scores in the HPX group were markedly greater than the vehicle group in each corresponding day (P<0.01, Figure 6A). In addition, brain infarct sizes were significantly reduced in the HPX group on the 7th day after MCAO (P<0.01, Figure 6B), which was consistent with the data of behavioral observations.

## Discussion

The aim of this study was to test the HPX expression in normal brain tissue and to determine the role of HPX during focal cerebral ischemia injury. We found that HPX positively expressed in several brain regions under physiological conditions, and the protein level of HPX was up-regulated at 24 h after middle cerebral artery occlusion. Further, intracerebroventricular administration of HPX in vivo dose-dependently decreased the ischemic penumbra area and improved neurological outcomes at 24 h after ischemia-reperfusion. These findings gave direct evidence of the neuroprotective function of HPX in focal cerebral ischemia injury, indicating its potential use in clinical treatment of ischemic stroke.

HPX is a plasma protein, which is recognized to serve a protective function by binding and transporting free heme, thus limiting the toxic effects that are mediated by free heme. Cytotoxic properties of free heme are thought to be involved in many complex cellular mechanisms, including the release of redox-active iron, production of superoxide and hydroxyl radicals; and peroxidation of membrane lipids [16]. A number of ischemia-reperfusion directed pathological processes are thought to be mediated by free heme oxidative stress pathways, including heme-dependent lung oxidative stress [17], liver reperfusion injury [18] and type 1 diabetes [19]. In vitro experiments, heme was shown to be toxic to both cortical neurons and astrocytes [10,20,21]. The primary defense for cells against heme toxicity is currently thought to be provided by a complex of heme with HPX, which has a very high affinity for heme [22]. The heme-HPX complex is taken up by cells through receptor-mediated endocytosis, which is then catabolized by the heme oxygenase isozymes (HO1 and HO2) [23]. Thus, HPX serves to regulate the balance between free heme and bound heme, and/or to regulate heme degradation. In this way HPX plays a cytoprotective role in cell survival, and pivotally does so in terms of short-term cellular defense as a powerful scavenger of free heme. It was reported that HPX was also synthesized locally by cells of CNS [6,9]. The present study further delineated the cellular localization of HPX in detail and found that HPX mainly expressed in cells of vascular system, neurons in limited brain regions, as well as astrocytes adjacent to blood vessels. These findings provide bases for the action of HPX in central nervous system.

Beneficial effects of HPX in CNS injury have been previously reported in transient middle cerebral artery occlusion [10]. This study found that HPX-null mice were more vulnerable to oxidative stress during cerebral ischemia injury than their wild-type counterparts, an observation which was consistent with a recent study in cultured neuronal cells, which showed that HPX decreased heme accumulation and catabolism [24]. Together, these evidences indicate that HPX might offer neuroprotective effects following ischemia-reperfusion injury. In their study they also found that the mRNA level of HPX was not increased after cerebral ischemia-reperfusion. However, they only examined at 96h, quite long time after the onset of reperfusion. HPX has been reported to be an atypical acute phase reactive protein [25,26], protein expression might have been temporarily changed in earlier phase. In the present study, we found that protein level of HPX in the ischemic penumbra was increased at 24h following ischemia-reperfusion injury as compared with the sham-operated group. Elevated HPX expression levels would attribute to a number of functionally important cytokines by immune cells activated during the process of stroke [27]. Inflammatory cytokines like IL-6, IL-1β and TNF-α were identified as potential promoters of the HPX gene [25,26].

It’s generally accepted that neurons and astrocytes in the ischemic penumbra were most vulnerable to heme-induced cytotoxicity. In present study we found that in comparison to the physiological conditions, the expression of HPX protein was significantly increased after cerebral ischemia, mainly in neurons and also in a small amount of astrocytes located in the subendothelial layer of the vessels. The cells that express augmented levels of HPX were located in the cerebral cortex, hippocampus, and striatum around the vicinity of the ischemic penumbra. Increased availability of HPX expressed in these local cells under ischemic condition combined with the protein constantly expressed in the vascular system, enabled HPX to deplete excess accumulation of overloaded heme generated by the ischemia-reperfusion process [28] in a relatively short period, thus avoid subsequent irreversible damage. This could be interpreted as the body’s endogenous protective mechanism to avoid suffering from ischemia-reperfusion injury, in which process HPX developed an important neuroprotective function. In our study, this function was further confirmed using an in vivo model of MCAO. We found that intracerebroventricular administration of HPX improved neurologic function and dampened infarct volumes in a dose-dependent manner following focal cerebral ischemia injury. Moreover, the neuroprotective effects sustained for 7 days after reperfusion. Taken together, these data provide novel insights that support the clinical utility of HPX in attenuating ischemia-reperfusion injury in CNS. Accumulated evidences [10,17,18,24,29] have proven the protective effect of HPX on different organs and cells. Our current findings are consistent with these studies and provide further evidence for the cytoprotective function of HPX.

Considering the poor efficacy, narrow treatment time-window, and the side effects of current therapeutic strategies in stroke, HPX exhibits promising superiority in improving the clinical management of stroke. Several challenges remain for this to be realized. One of the more challenging obstacles is to identify a clinically acceptable means of delivering HPX to brain cells. Due to its high molecular weight, it is challenging to adopt HPX in clinical practice because it cannot readily transit the blood–encephalic barriers to carry out its neuroprotective effect. This is exemplified by the fact that low levels of HPX, with a mean concentration of 2.6 mg/l and a range of 1.8–3.4 mg/l [30,31] in the cerebrospinal fluid would be insufficient to cope with the quantities of heme released during ischemia reperfusion. Additionally, the mechanisms underlying the neuroprotective effects of HPX in stroke remain controversial. Fully understanding the mechanisms of action of HPX will provide a hypothetical foundation to optimize therapeutic strategies and formulate an international standard for clinical practice. In conclusion, our study has provided novel information whereby endogenous cytoprotective mechanisms could be activated by HPX in an attempt to clinically manage ischemia stroke.

## Conclusion

In this study we found that HPX is mainly expressed in vascular system and neurons, and is increased in penumbra area after focal cerebral ischemia-reperfusion injury. Administration of the HPX induces an evident and long-lasting neuroprotective effect. These results indicate that HPX might be developed as a potential agent for treatment of ischemic stroke.

## Abbreviations

HPX: Hemopexin; I/R: Ischemia/reperfusion; CNS: Central nervous system; MCAO: Middle cerebral artery occlusion; TBST: Tris-Buffered Saline Tween-20.

## Competing interests

No conflict of interest exits in the submission of this manuscript.

## Authors’ contributions

LX and HD contributed to designing the study. BD and MC contributed to performing the Western blotting, immunohistochemical procedures. ZF and HW contributed to writing the manuscript. FZ and GL contributed to data analyze. All authors read and approved the final manuscript.
